# Tax on Patent Medicines

**Published:** 1904-08

**Authors:** 


### TAX ON PATENT MEDICINES.

THE Georgia Legislature has passed a bill requiring dealers in the proprietary nostrum so widely advertised, called Peruna, to pay a license of $200. This preparation is alleged to contain a high percentage of alcohol, and it was the object of the bill to protect the unwary public from acquiring the alcoholic habit unwittingly from taking the nostrum. A list has been published from time to time of proprietary preparations which contain a proportion of alcohol sufficiently large to constitute a danger to the public. Several magazines and newspapers have placed such preparations on the list of unacceptable advertisements, and others are following their example. The W. C. T. U. has been active in the matter and has shown its intention of warning the public against the treacherous and seductive character of these secret remedies. The beneficial results claimed to have been obtained by the users of these compounds are ascribed to the exhilarating influence of the alcohol contained, which, in some of them, exists in a percentage almost equal to that of sherry, and very much greater than that of beer and the light wines.

These are indications that the people are being protected by others than their traditionary guardians, the doctors, and it is to be hoped that the crusade will extend to include the suppression or restriction of all such advertised remedies as contain ingredients hurtful or enslaving, such as catarrh snuffs containing cocaine and soothing-syrups with opium. Advertising is the life of the proprietary medicine, and without it it rapidly falls into oblivion. The habit of self-diagnosis and self-administered therapeutics so prevalent among the laity is a powerful coadjutor of the quack remedy. If the newspapers and magazines would withdraw their advertising support and the public warned that they were warming a snake, the days of Peruna and its confreres would be numbered. The public is not such a fool. What it needs is more light in dark places.

WE take pleasure in announcing the addition to the editorial staff of Dr. J. N. LeConte. Dr. LeConte is admirably fitted for medical journalistic work, as he is a man of broad and liberal views, with a mind well stored with general and professional information. He is sprung from a highly distinguished Georgia family, and is the nephew of Joseph LeConte of California, the eminent geologist.

The JOURNAL-RECORD congratulates itself upon securing the collaboration of Dr. LeConte, and is confident that its readers will appreciate his services.

---

THE new Hospital for Incurables under the management of the King's Daughters, and located at Woodward avenue and South Boulevard, was formally opened with appropriate ceremonies on July 18. There are five wards, most of them memorial, for the accommodation of private pay patients as well as for indigent sufferers from incurable diseases. Dr. S. A. Visanska is the physician in charge, and much of the credit for securing the new building is due to his zeal and enthusiasm.

---

A COMMITTEE has been appointed by the Atlanta Society of Medicine, consisting of Dr. E. G. Jones, Dr. J. S. Todd and Dr. W. E. Wilmerding, for the purpose of collecting evidence against alleged illegal practitioners in Atlanta, in order to present their names to the grand jury. It is hoped that many true bills will be found and that the city will be ridded of the swarm of quacks now infesting it.

---

THE Legislature has passed on the appropriation of $10,000 for the State Board of Health. An effort was made to cut down the appropriation one half but failed. There is, of course, no more important appropriation than this, and even though small and inadequate it will no doubt considerably facilitate the work of the board.

